# Chronic Exposure to Perfluorooctane Sulfonate Induces Behavior Defects and Neurotoxicity through Oxidative Damages, *In Vivo* and *In Vitro*

**DOI:** 10.1371/journal.pone.0113453

**Published:** 2014-11-20

**Authors:** Na Chen, Jia Li, Dan Li, Yongsheng Yang, Defu He

**Affiliations:** 1 Lab of Toxicology, School of Ecological and Environmental Sciences, East China Normal University, Shanghai, China; 2 Shanghai Key Lab for Urban Ecological Processes and Eco-Restoration, East China Normal University, Shanghai, China; Northeastern University, United States of America

## Abstract

Perfluorooctane sulfonate (PFOS) is an emerging persistent pollutant which shows multiple adverse health effects. However, the neurotoxicity of PFOS and its mechanisms have not been fully elucidated. Using a combination of *in vivo* and *in vitro* methods, the present study provides a detailed description of PFOS-induced neurotoxicity. Results showed that the median lethal concentration of PFOS was 2.03 mM in *Caenorhabditis elegans* for 48 h exposure. 20 µM PFOS caused decrease of locomotor behaviors including forward movement, body bend and head thrash. Additionally, PFOS exposure reduced chemotaxis index of *C. elegans*, which indicates the decline of chemotaxis learning ability. Using green fluorescent protein (GFP) labelled transgenic strains, we found that PFOS caused down-regulated expression of a chemoreceptor gene, *gcy-5*, in ASE chemosensory neurons, but did not affect cholinergic neurons and dopaminergic neurons. In SH-SY5Y cells, 48 h exposure to 25 µM and 50 µM PFOS induced cell damage, apoptosis and the reactive oxygen species (ROS) generation. PFOS caused significant increases of lipid peroxidation and superoxide dismutase activity, but an actual decrease of glutathione peroxidase activity. Furthermore, antioxidant N-acetylcysteine rescued cells from PFOS-induced apoptosis via blocking ROS. Our results demonstrate that chronic exposure to PFOS can cause obvious neurotoxicity and behavior defects. Oxidative damage and anti-oxidative deficit are crucial mechanisms in neurotoxicity of PFOS.

## Introduction

Perfluorinated compounds (PFCs) are a family of fluorine-containing chemicals with properties that make materials stain resistant. PFCs have been widely used for more than 50 years, mainly as components of fire retardants, lubricants, adhesives, insecticides, pharmaceuticals [1]. Perfluorooctane sulfonate (PFOS) is the principal chemical or metabolite of PFCs. As PFOS is characterized by persistence and bioaccumulation, high levels of PFOS has been detected in the environment, as well as in organisms [2]–[5]. For example, the median value of PFOS in the serum of non-occupationally exposed humans was 18.8 ng/L, with a range of 8.1 to 150.7 ng/L [6]. A range of 0.1 to 100 ng/L PFOS was generally found in surface water, and concentrations of up to 600 ng/L had been reported in rivers near fluorochemical manufacturing facilities [7]. As emerging persistent organic pollutants, PFOS and its salts were officially added into the list of the “Stockholm Convention” in 2009. PFOS has been restricted, or even banned to product in many countries, but it is still ubiquitous in the environment. The potential health risks of PFOS have drawn great concerns.

Perfluorooctane sulfonic acid has been shown multiple adverse health effects, including developmental toxicity, immunotoxicity, hepatotoxicity, endocrine disruption, and neurotoxicity [8]. PFOS is mostly accumulated in liver, serum, and brain in mammals [9]. The pharmacokinetics of PFOS is nonlinear, and the half-life for elimination from the body varies between species [10], [11]. The average serum half-life of PFOS in humans is 5.4 years [12]. As PFOS can cross the blood-brain barrier [13], its potential neurotoxicity has received growing attentions. Previous studies have revealed that neonatal exposure to PFOS effected normal brain development and caused neurobehavioral defects in mice [14], [15]. Additionally, exposure to PFOS caused a deficit in spatial memory without disturbing motor function in mice [16]. However, the neurotoxicity of PFOS and its mechanisms have not been fully elucidated.

*C. elegans* is an emerging model in environmental toxicology. It has several advantages for model animal, for example, its small size and short life cycle. *C. elegans* contains 302 neurons and its neuronal lineage is fully described [17]. Additionally, increasing evidence shows that *C. elegans* is similar with mammals, both on the genetic and the physiological level [18]. Thus, results from *C. elegans* have the potential to predict possible effects in higher animals.

In the present study, the chronic exposure of low dosage PFOS was conducted in *C. elegans*. Behavior changes including locomotor and chemotaxis learning behaviors were assayed. Neurotoxic effects were respectively investigated on cholinergic neurons, dopaminergic neurons and ASE chemosensory neurons in transgenic nematode. Furthermore, PFOS was exposed to the human neuroblastoma cells, SH-SY5Y cells. Cellular viability, apoptosis level, reactive oxygen species, and relative enzymes were respectively determined. Our aim was to investigate the neurotoxicity of PFOS and its mechanism through a combination with of *in vivo* and *in vitro* methods.

## Materials and Methods

### Reagents

Perfluorooctanesulfonic acid potassium salt (PFOS), Dimethylsulfoxide (DMSO), 3-(4,5-Dimethylthiazol-2-yl)-2,5-diphenyltetrazolium bromide (MTT) were purchased from Sigma (St. Louis, MO, USA). PFOS was dissolved in DMSO and stored at 4°C [19]. Dulbecco's modified Eagle's medium (DMEM), Fetal bovine serum (FBS) were purchased from Hyclone Company (USA). Antioxidant enzyme detection kits (SOD, GSH-PX), ROS and MDA detection kits were purchased from Nanjing Jiancheng Bioengineering Institute (Nanjing, China). Annexin V-FITC apoptosis detection kit was purchased from Beyotime Institute of Biotechnology (Jiangsu, China). Agar, cholesterol and other chemicals were obtained from Sinopharm Chemical Reagent Co., Ltd (Shanghai, China).

### *C. elegans* strains and culture

Wild type N2 *C. elegans* and three transgenic strains including *OH2871* [*gcy-5::gfp*; Expression of GFP in both ASEL and ASER neurons] [20], *OH10819* [*unc-17::gfp*; GFP expressed in all cholinergic neurons] [21], and *BZ555* [*Pdat-1::gfp*; Bright GFP observable in dopamine neuronal soma] [22] were donated by Caenorhabditis Genetics Center (Twin Cities, USA). *C. elegans* were maintained on nematode growth medium (NGM) plates fed with Escherichia coli OP50 at 20°C [23]. Adult nematodes were transferred into tubes by washing plates with M9 buffer, and then were dissolved by a bleaching buffer (0.45 N NaOH, 2% HClO). The eggs were collected and hatched on the plates without food, then synchronized for further experiments [24].

### Lethality

PFOS solutions were prepared in DMSO (final concentration was 1%). 1% DMSO was not toxic to nematodes as determined by viability assays (data not shown). *C. elegans* were exposed to a series of concentrations of PFOS (0.01, 0.1, 0.5, 1, 5 and 10 mM) in the 24-well plates [25]. Each well contained 1 ml PFOS solutions, 150 mM fluorodeoxyuridine (to prevent eggs from hatching) and 50 age-synchronized nematodes. 1% DMSO was utilized in the control well. After 48 h exposure, dead nematodes were identified when they did not respond to stimulating their body. Three replicates were set up for each group. Then the median lethal concentration (LC_50_) of PFOS was determined by nonlinear regression analysis (Graphpad Prism software).

### Locomotor behavior assay

Locomotor behavior was assessed including basic movements, head thrash, body bend and crawling speed. After 48 h exposure, nematodes were respectively transferred into fresh NGM plates. Video images were recorded and analyzed off-line. Then the number of basic movements including forward movement, backward movement and omega turn were counted for 1 min. Head thrashes and body bends were respectively counted for 1 min and 30 s [26], [27]. The crawling speed was analyzed by software Wormlab [28]. Triplicate tests were performed.

### Chemotaxis behavior assay

According to the previous study [29], the chemotaxis assay was carried out to determine learning ability. After 48 h exposure, nematodes were washed three times with M9 buffer, and then transferred to NGM plates for training. Shortly before the chemotaxis assay, approximately 100 nematodes were starved for 3 h on NGM plates with NaCl or without NaCl. Animals were transferred to the starting point of testing plates that were prepared in advance. After 30 min, by anesthetization of NaN_3_, numbers of nematodes at the section with NaCl and that without NaCl were respectively counted, then the chemotaxis index (CI) was calculated by Saeki' method [29]. Tests were performed in triplicate.

### Fluorescent images of neurons

Transgenic nematodes *OH2871* was utilized to investigate the toxic effects of PFOS on chemosensory ASE neurons including the left ASE neuron (ASEL) and the right ASE neuron (ASER) [20]. GFP transgenic nematodes, *OH10819* and *BZ555* were respectively used to check the toxic effects on cholinergic neurons and dopaminergic neurons [21], [22]. After 48 h exposure, nematodes were washed three times with M9 buffer and mounted onto agar padded glass slide. Images of immobilized nematodes were captured by a fluorescence microscope (LEICA DM400 B). Intensities of fluorescent puncta for ASE, cholinergic neurons and dopaminergic neurons were respectively examined in at least 30 nematodes. Fluorescence intensity was quantified by Image J software.

### SH-SY5Y cell culture and exposure

SH-SY5Y cells (a subline of the neuroblastoma cell line SK-N-SH) were grown in DMEM medium containing 10% FBS at 37°C in 5% CO_2_. Cells were seeded on flat bottom 96-well plates at 1000 cells/well, and maintained in a humidified incubator containing 5% CO_2_ at 37°Cfor 24 h, then incubated with PFOS solutions (5, 25, 50 or 100 µM) for further assays. PFOS were dissolved in DMSO, which was used at the same final concentration of 1‰ (v/v). For the ROS and apoptosis inhibition study, cells were treated with 10 mM N-acetylcysteine (NAC) for 24 h before exposure to PFOS.

### Viability assay

Cell viability was determined by MTT assay. After PFOS (0, 5, 25, 50 and 100 µM) exposure for 48 h, 20 µl MTT (5 mg/mL) was added to each well and kept incubating. 4 h later, 150 µL DMSO was added to each well after the medium was removed. The optical density of the MTT color reaction was measured with a microplate reader (Infinite M200, Tecan, Australia) at 490 nm. Cell viability was shown as percentage of the optical density in comparison with the control.

### Analysis of apoptosis

After exposure for 48 h, cells were harvested and re-suspended in PBS at a concentration of 1×10^6^ cell/ml. After centrifuged at 1000 g for 5 min, 195 µL FITC-conjugated Annexin V binding buffer and 5 µL of Annexin V-FITC were added. Following gentle vortex, the mixture was incubated for 15 min at room temperature in the dark. After centrifuged at 1000 g for 5 min, 190 µL FITC-conjugated Annexin V binding buffer and 10 µL propidium iodide (PI) were added. Following gentle vortex, the sample was analyzed using flow cytometer (Becton Dickinson, Mountain View, CA). The percentages of apoptotic cells were determined.

### Detection of reactive oxygen species (ROS)

After exposure, culture medium was removed and 200 µl DCFH-DA (which was diluted by serum-free DMEM) was added to each well, and then cells were kept incubating for 20 min. Supernatant were removed and cells were washed with PBS for three times, in order to eliminate DCFH-DA which didn't enter into cells. Finally, the optical density of each sample was immediately measured in an ELISA microplate reader (Infinite M200, Tecan, Australia) at 488 nm.

### Assay of MDA (Malondialdehyde) contents and antioxidant enzymes

After exposure, cells in each well were moved to test tube, centrifuged at 1000 r/min for 10 min at 4°C, and washed with PBS for three times. 500 µL PBS was added to each tube. Then cells were subjected to ultrasonication (power of 500 W) for 5 min in ice-water bath (pause 30 s and keep 30 s). Total protein concentration was examined by BCA Protein Assay Kit (Beyotime Institute of Biotechnology). MDA level, superoxide dismutase (SOD) and glutathione peroxidase (GPX) activities were measured using relevant assay kits (Nanjing Jiancheng Bioengineering Institute).

### Statistics

All data were expressed as mean ± Standard Error of Mean (SEM). One-way ANOVA and Duncan's test was carried out by SPSS 16.0. A p-value of less than 0.05 was considered significant.

## Results

### Lethal effects of PFOS in *C. elegans*

**Figure 1:**
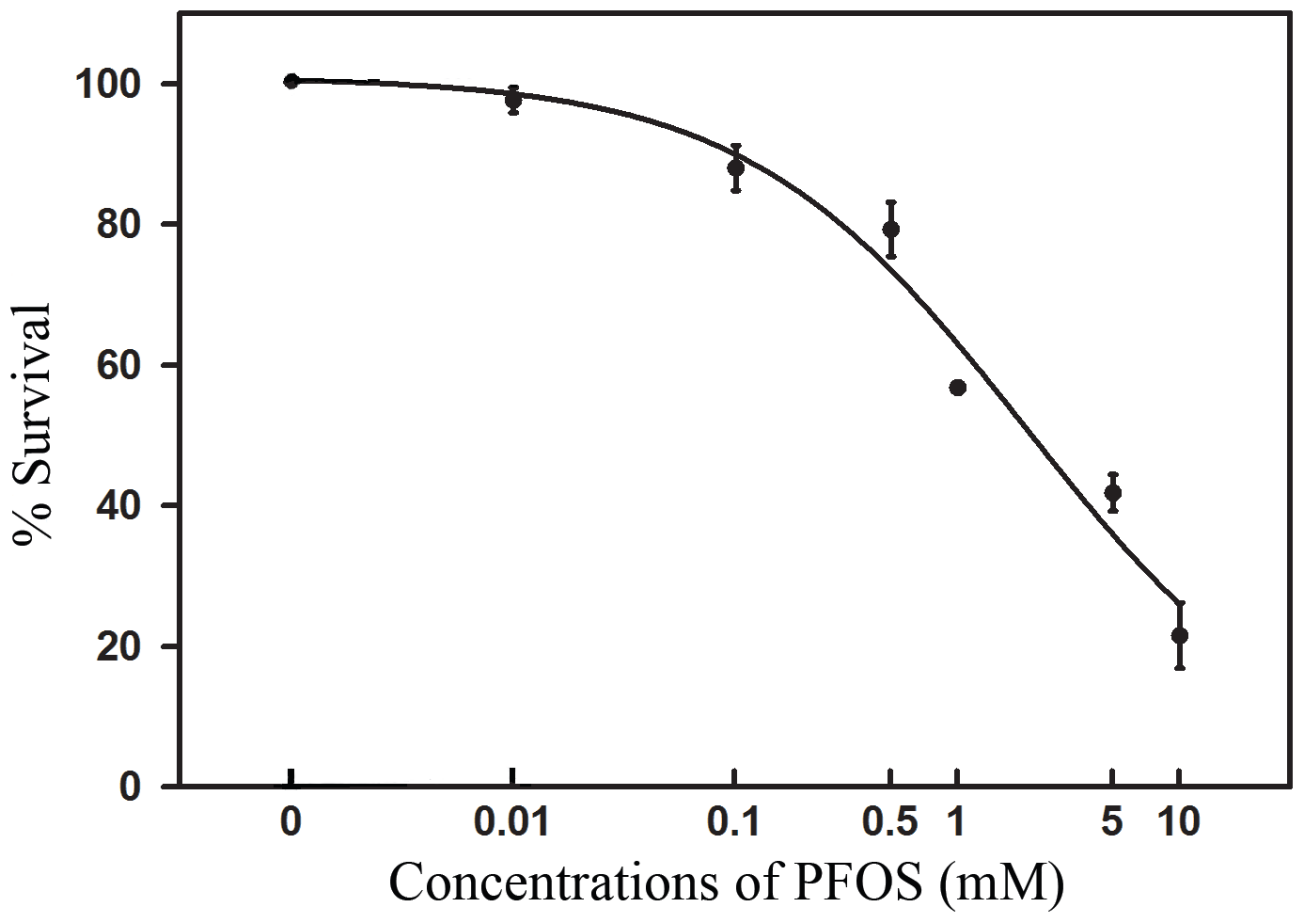
Survival percentages of *C. elegans* after exposed to PFOS for 48 h. Data are expressed as mean ± SEM of three separate experiments.

As shown in Figure 1, survival percentages of *C. elegans* decreased in a dose dependent manner. The best-fit median lethal concentration (LC_50_) of PFOS is 2.03 mM for 48 h exposure (95%CI = 1.65–2.50; R^2^ = 0.97).

### Effects of PFOS on locomotor behaviors in *C. elegans*

**Figure 2:**
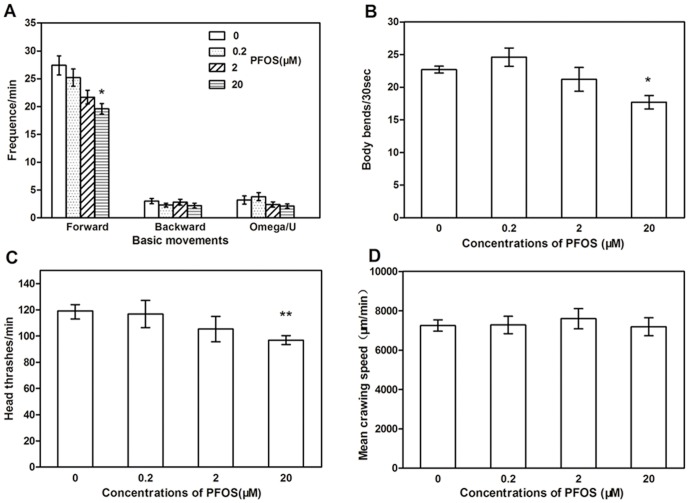
Effects of PFOS exposure on locomotor behaviors in *C. elegans*. (A)–(D) show respectively the change of basic movements (A), head thrash (B), body bend (C) and crawling speed(D) after 48 h exposure to PFOS. *p<0.05 and **p<0.01, compared with the control.

*C. elegans* was exposed to 0.2 µM, 2 µM, or 20 µM PFOS for 48 h. Then basic movement, body bends, and head thrashes were respectively examined [27]. Results show that 20 µM PFOS caused a significant reduction in frequency of forward movement compared to the control worms (*p*<0.05), but not obvious change in the frequency of backward movement and omega turn (Figure 2A). A substantial decrease in body bends occurred after exposed to 20 µM PFOS (*p*<0.05, Figure 2B). Otherwise, 20 µM PFOS exposure also caused a significant decrease of head thrash frequency in *C. elegans* (*p*<0.01, Figure 2C). We did not observe changes of crawling speed between the exposed groups and the control (Figure 2D).

### Effects of PFOS on chemotaxis learning in *C. elegans*

**Figure 3:**
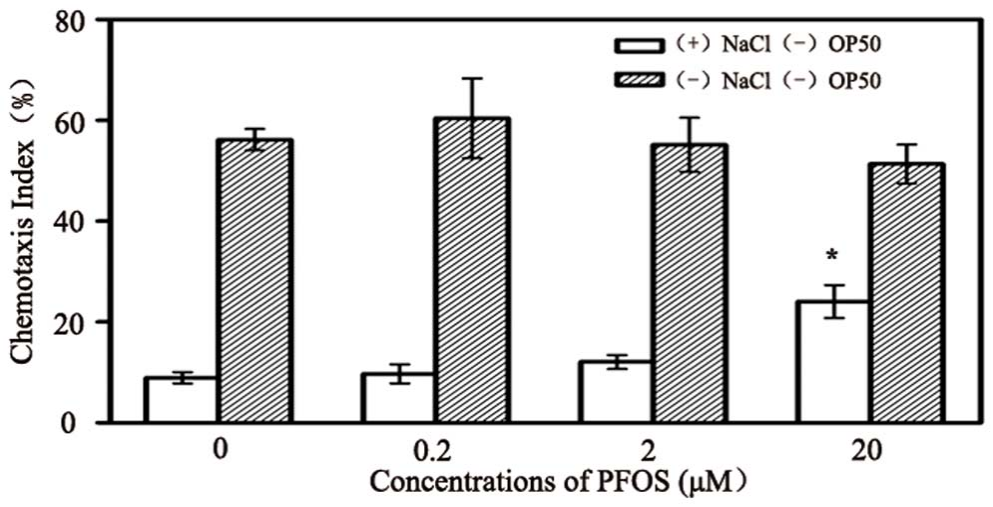
Effects of PFOS treatment on chemotaxis index in *C. elegans*. Escherichia coli OP50 were used as food for *C. elegans*. Data are expressed as mean ± SEM. *p<0.05 compared with the control.

Chemotaxis plasticity is an emerging approach to assay learning ability in *C. elegans* [29]. Nematodes present chemotaxis plasticity when combined food with NaCl. According to the previously described [29], *Caenorhabditis elegans* shows a chemotactic behavior toward NaCl. However, it learns to avoid NaCl after prolonged exposure to NaCl under starvation conditions, which is called salt chemotaxis learning. So nematodes will be attracted by NaCl after starved on the plate without NaCl for training. The chemotaxis index was 55.8±4.2% for these worms in this study. If starved on the plate with NaCl for training, these worms showed relatively low chemotaxis index (Figure 3, the control group). We further investigated the effects of PFOS exposure on chemotaxis learning. After exposure to 0.2 and 2 µM PFOS, chemotaxis index was similar to the control (Figure 3). But 20 µM PFOS caused a significant increase of chemotaxis index when starved in NaCl condition (Figure 3). It demonstrates that exposure to 20 µM PFOS affected chemotaxis behavioral plasticity in nematodes.

### PFOS affected ASE sensory neurons in *C. elegans*

**Figure 4:**
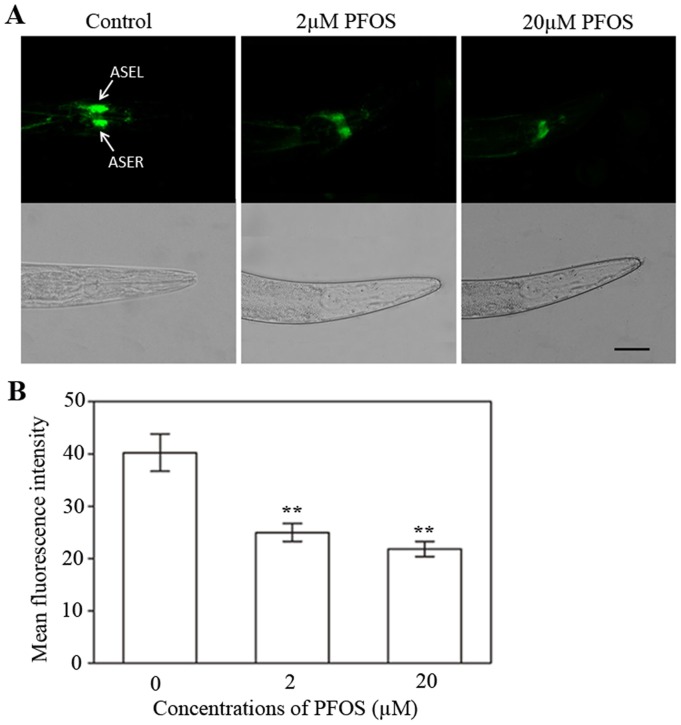
Effects of chronic PFOS exposure on ASE sensory neurons in transgenic *C. elegans* strain *OH2871*. (A) GFP expression pattern in ASEL and ASER neurons (arrows) of transgenic strain *OH2871* in the control or PFOS-exposed condition. Upper parts are fluorescent images; down parts are differential interference contrast images. (B) Effects of PFOS exposure on fluorescent intensities of cell bodies in ASE sensory neurons in transgenic strain *OH2871*. Data are expressed as mean ±SEM. ** *p*<0.01, compared to the control. Bar  = 50 µm.

A pair of ASE sensory neurons plays key roles in chemotaxis behavior of *C. elegans*. In transgene strain *OH2871*, both ASEL and ASER showed expression of *gcy5-gfp* (Figure 4A, control). So fluorescent intensity of GFP was usually used for cell fate markers for ASE neurons [30], [31]. After 48 h treatment with 2 µM and 20 µM PFOS, the size of fluorescent puncta was obviously decreased in ASE sensory neurons (Figure 4A). In addition, PFOS exposure significantly suppressed fluorescent intensities of cell bodies in ASE sensory neurons compared to the control (*p*<0.01, Figure 4B). However, we did not observe obvious changes of cholinergic neurons or dopaminergic neurons of *C. elegans* after exposed to PFOS (Figure S1).

### Effects of PFOS on SH-SY5Y cells

**Figure 5:**
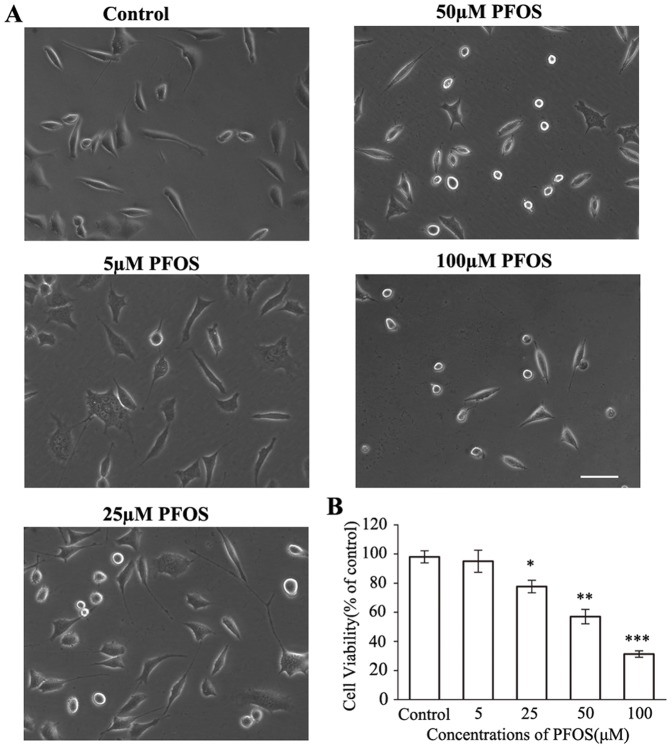
Effects of PFOS exposure on SH-SY5Y cells. Cells were treated with a series of concentrations of PFOS for 48 h followed by the MTT assay. (A) Cells were photographed under light microscopy, bar  = 20 µm. (B) Cell viability of control and experimental groups. Data are expressed as mean ± SEM of four separate experiments. *p<0.05, ** *p*<0.01 and *** *p*<0.001, compared to the control.

We further investigated the neurotoxicity of PFOS and its mechanisms in human neuroblastoma SH-SY5Y cells. After 48 h exposure, cell morphology and viability were assayed. In the exposed groups, cells were distributed sparsely, many cells represented unremarkable colonies, bad refraction, fracture and shrinking of neurite (Figure 5A). After high concentration (50 µM and 100 µM) exposure, serious damage occurred, for example, some of cell axons disappeared, and cell bodies crimpled. Results showed that PFOS caused cell viability decreased in a dose dependent manner (Figure 5B).

### PFOS induced apoptosis in SH-SY5Y cells

**Figure 6:**
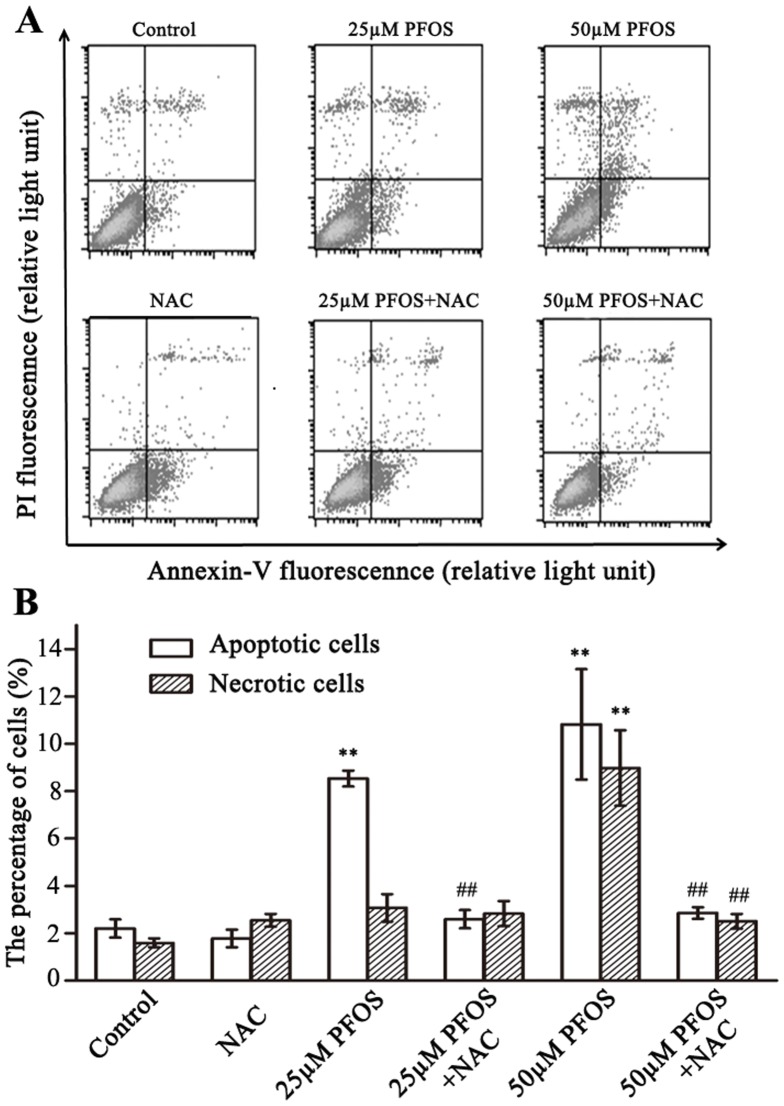
PFOS exposure induced apoptosis in SH-SY5Y cells. (A) With (upper) or without pretreatment of NAC (down), cells were exposed to DMSO or PFOS (25 and 50 µM) for 48 h and then apoptosis was analyzed by flow cytometer using Annexin-V and PI stain. X-axis represents increasing Annexin-V fluorescennce (relative light unit) and Y-axis represents increasing PI fluorescennce (relative light unit). (B) The bar graph represents the percentage of apoptotic cells and necrotic cells. Data are expressed as mean ± SEM. ** *p*<0.01compared to the DMSO control, ^##^ *p*<0.01 compared to only PFOS treated groups.

Flow cytometry showed that apoptotic cells significantly increased after exposure to 25 µM or 50 µM PFOS (*P*<0.01, Figure 6). Pretreatment with NAC, a potent thiol antioxidant, rescued cells from PFOS-induced apoptosis, with the percentages of apoptotic cells respectively decreased from 8.53% to 2.59%, and from 10.82% to 2.85% (Figure 6B).

### PFOS-induced cytotoxicity is mediated by ROS

**Figure 7:**
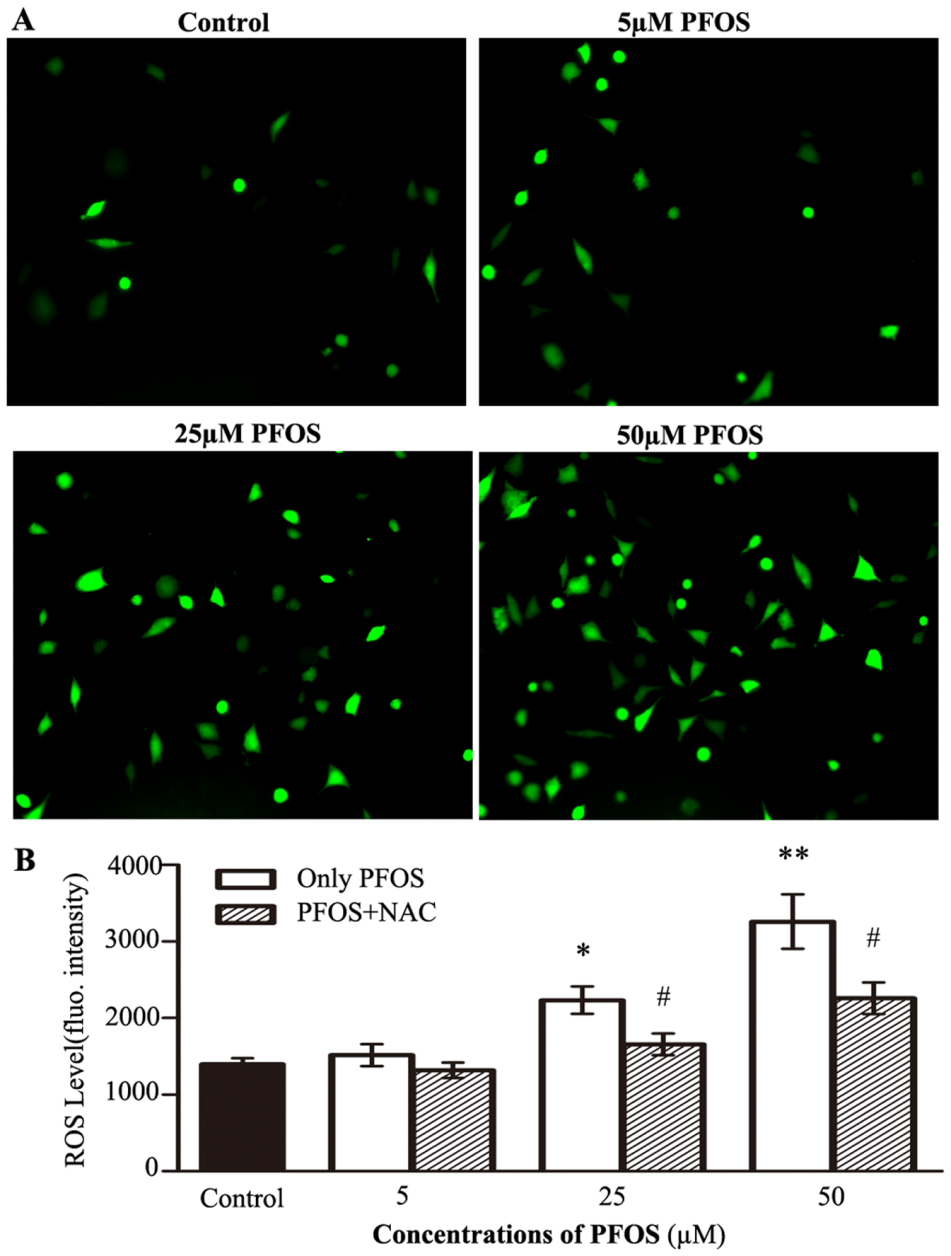
PFOS induced ROS generation in SH-SY5Y cells. (A) Cells were photographed under fluorescence microscope, bar  = 20 µm. (B) The bar graph represents the ROS level of different PFOS treatments. Data are expressed as mean ± SEM. *p<0.05 and *p*<0.01compared to the DMSO control, ^#^ *p*<0.05compared to only PFOS treated groups.

We further examined ROS production in the neuroblastoma cells. Utilizing DCFH-DA, the intensity of intracellular ROS was obviously labeled by fluorescence (Figure 7A). After 48 h exposure to 25 µM or 50 µM PFOS, ROS level was significantly elevated (Figure 7A, B). Furthermore, the increase of ROS was blocked by the pretreatment of 10 mM NAC (Figure 7B). It demonstrated that PFOS was an effective stimulant of ROS generation in the cells.

**Figure 8:**
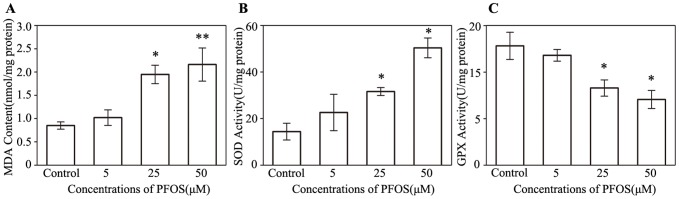
Effects of PFOS on oxidative and anti-oxidative enzymes in SH-SY5Y5 cells. MDA content (A), SOD activity (B) and GPX activity (C) were measured after treatments with PFOS. *p<0.05 and ** *p*<0.01 compared to the control.

As MDA is an indicator for lipid peroxidation, we further examined MDA content in cells. Results showed that PFOS exposure caused the increase of MDA activity in a dose-dependent manner (Figure 8A). 25 µM and 50 µM PFOS induced over 2 fold increase of MDA content. In the oxidation system, SOD and GPX play an important role in radical scavenging to prevent cell from oxidative damage. Our results showed that 25 and 50 µM PFOS caused significant increases of SOD activity (Figure 8B). Nevertheless, GPX activity was significantly decreased in 25 µM and 50 µM PFOS groups compared to the control (Figure 8C).

## Discussion

As an emerging persistent pollution, PFOS has a global occurrence in the environment and biology [32]–[34]. The health risks associated with PFOS has received wide concerns. Our results showed the obvious lethal effects of PFOS in *C. elegans*. It indicates the ecological risks of chronic PFOS exposure, which is consistent with previous studies on other test organisms [35]–[38]. Furthermore, the neurotoxic effects were examined after *C. elegans* were exposed to 0.2–20 µM PFOS. Endpoints of basic movement frequency, body bending, head thrashing, and crawling speed were used to evaluate changes in locomotor behavior. Significant decreases in body bends, head thrashes, and forward movement frequency were demonstrated. It suggests the obvious locomotor deficits induced by PFOS. There is limited, but growing evidence linking PFOS exposure to neurobehavioral toxicity [39]–[41]. Neonatal exposure to high dose PFOS (21 mmol/kg/d) caused abnormal spontaneous behavior, increased motor activities, and reduced habituation in mice [41], [42]. Otherwise, adult animals exposed to PFOS had shown only slight neurobehavioral effects [15], [16], [39]. In the present study, the obvious neurobehavioral changes of *C. elegans* were the response to chronic exposure (several life stages including L2, L3 and L4 stages on sublethal dosage levels).

In chemotaxis learning, *C. elegans* navigated NaCl in an experience-dependent manner [43]. Our results showed that PFOS exposure could affect chemotaxis plasticity and reduce learning ability in nematodes. It suggests that chronic exposure to PFOS can result in multiple neurobehavioral defects in animals. ASE sensory neurons, cholinergic neurons and dopaminergic neurons have been identified to be relative to chemotaxis behaviors in *C. elegans* [44]. In transgenic strain *OH10819* and *BZ555*, UNC-17::GFP and PDAT-1::GFP were respectively used as fluorescent markers for cholinergic neurons and dopamine neurons [21], [22], [30], [45]. However, worms expressing GFP-labeled cholinergic neurons or dopamine neurons did not show noticeable changes after PFOS exposure. It indicates that PFOS has no impact on the function of cholinergic neurons and dopamine neurons in *C. elegans*.

In *C. elegans*, *gcy-5* is a key gene which encodes chemoreceptors in the ASE chemosensory neurons [20], [44]. GCY-5 localizes to sensory endings exclusively in ASEL and ASER chemosensory neurons [44], [46]. Therefore, GCY-5::GFP could be used as a specific fluorescent marker for the ASE sensory neurons [30]. Our results showed that PFOS exposure caused the decrease in the size of fluorescent puncta, and reduced fluorescence intensity of GCY-5::GFP in ASE sensory neurons. It indicates that chronic exposure to PFOS can cause down-regulated expression of *gcy-5*, thus affect the function of ASE chemosensory neurons. For the first time, we found differential effects of PFOS among ASE sensory neurons, cholinergic neurons and dopamine neurons *in vivo*. However, further study should be conducted to shed light on the underlying mechanisms.

In SH-SY5Y cells, our results show that PFOS induced cell damage and caused a decrease in cell viability. It is in line with previous studies in N9 cells and HepG2 cells [47], [48]. In addition, PFOS-induced apoptosis was raised in a dose-dependent manner. Previous studies have also shown the PFOS-induced apoptosis in lung cancer A549 cells and cerebellar granule cells [49], [50]. These, along with our results, indicate that apoptosis is an important factor in the cytotoxicity of PFOS.

Numerous studies have shown that environmental contaminants produced free radicals and altered the antioxidants level [51]–[53]. The accumulation of oxidative damage to biomolecules has been implicated in the pathogenesis of neurodegenerative diseases [54]. Moreover, increased oxidative stress has been related to severely impair learning behavior, and modestly reduced motor activity [55], [56]. Our results show that the intracellular ROS level was significantly increased after cells were exposed to PFOS. Pretreatment of antioxidant N-acetylcysteine blocked PFOS-induced ROS generation, and then inhibited the apoptosis in SH-SY5Y cells. It indicates that intracellular ROS is important initiating signaling event of the PFOS-induced cytotoxicity. Furthermore, we observed that PFOS induced a decrease in GSH level, and an increase in MDA level and SOD in a dose-depend manner. It is consistent with another description in lung cancer A549 cells [50]. According to previous studies, the level of MDA reflects the degree of radical lipid peroxidation in cells. GSH is a vital intra- and extracellular protective antioxidant against oxidative stress. SOD is an important antioxidant enzyme that could counteract oxidative damage [52], [53]. Our results indicate that the neurotoxicity of PFOS is mediated, at least in part, by an oxy radical mechanism involving overproduction of ROS and down regulation of certain key antioxidant enzymes such as GPX. Both events would lead to oxidative stress in the PFOS-exposed cells.

In summary, the present study showed that chronic exposure to PFOS caused decrease of locomotor behaviors and defects in chemotaxis learning activity in *C. elegans*. PFOS exposure induced down-regulated expression of *gcy-5*, and affected function of ASE sensory neurons, but did not affect cholinergic neurons and dopamine neurons. It demonstrates PFOS caused differential effects on neurons *in vivo*. In SH-SY5Y cells, we revealed that PFOS-induced cytotoxicity was mediated by ROS and apoptosis. PFOS caused increase of MDA and SOD activity, but an actual decrease of GPX activity. Oxidative damage and anti-oxidative deficit are critical mechanisms in neurotoxicity of PFOS.

## Supporting Information

Figure S1 **Effects of PFOS exposure on cholinergic neurons and dopaminergic neurons in** ***C. elegans*** **.** The fluorescent images represent GFP expression pattern in cholinergic neurons of transgenic *C. elegans* strain *OH10819* (A) and dopaminergic neurons of transgenic strain *BZ555* (C) after exposed to DMSO, 2 or 20 µM PFOS for 48 h, bar  = 50(A), 100 µm(C), respectively. The bar graphs show fluorescence intensity of GFP expression in cholinergic neurons (B) and dopaminergic neurons (D). Data are expressed as mean ± SEM of four separate experiments.
(TIF)

File S1 **This file contains the data for the project.**
(XLS)
