# Supplementary figures and images for: Chronic Exposure to Perfluorooctane Sulfonate Induces Behavior Defects and Neurotoxicity through Oxidative Damages, In Vivo and In Vitro
Source: PLoS One. 2014 Nov 20;9(11):e113453. doi: 10.1371/journal.pone.0113453 (PMC4239059; doi:10.1371/journal.pone.0113453)

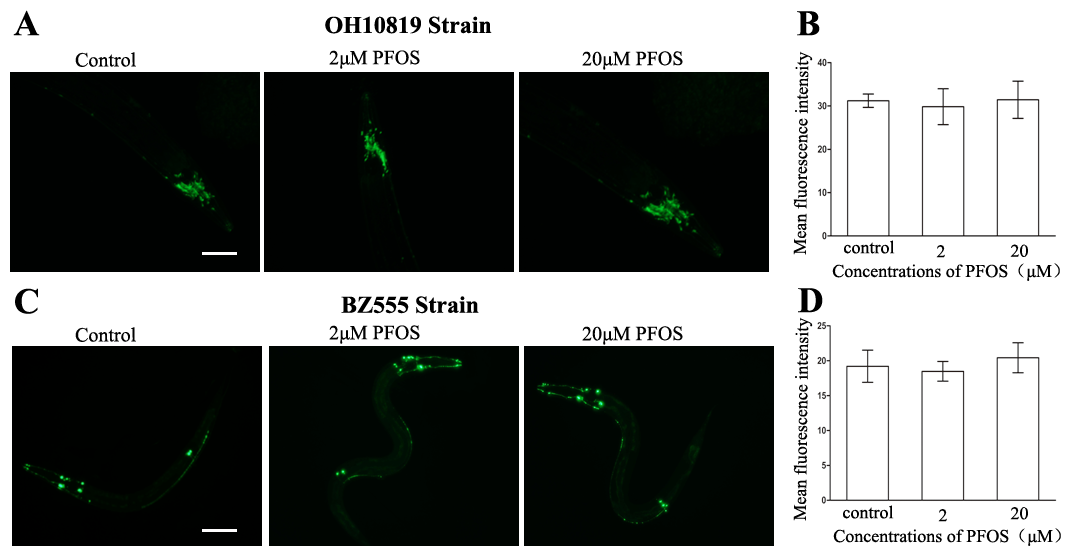

Supplement: Figure S1 — Effects of PFOS exposure on cholinergic neurons and dopaminergic neurons in C. elegans . The fluorescent images represent GFP expression pattern in cholinergic neurons of transgenic C. elegans strain OH10819 (A) and dopaminergic neurons of transgenic strain BZ555 (C) after exposed to DMSO, 2 or 20 µM PFOS for 48 h, bar = 50(A), 100 µm(C), respectively. The bar graphs show fluorescence intensity of GFP expression in cholinergic neurons (B) and dopaminergic neurons (D). Data are expressed as mean ± SEM of four separate experiments. (TIF) [file pone.0113453.s001.tif]
